# Annotations

**Published:** 1895-03-16

**Authors:** 


### Annotations.

### The Right Diet for John Bull.

A lay contemporary expresses some gratitude to The Hospital for protesting against too much monotony and spareness in eating and drinking. This is a question of really first-rate importance, seeing that Great Britain is largely what she is by virtue of what she eats and drinks. A late eminent physician of Scottish extraction, during the earlier years of his practice in London, used to have a rule of this kind in his mind: "Pull down the diet to the level of the stomach's capacity." That sounded excellently well in theory. In course of time, however, he found, as all competent doctors have found, that it does not work well in practice. A somewhat converse rule is infinitely better, at any rate for Londoners, and English people generally, however well "modified starvation" may be appreciated north of the Tweed. The better rule reads thus: "Raise up the capacity of the digestive organs by sound treatment until they can thoroughly digest sufficient for all the most liberal necessities of the body and brain." The former rule, of lowering diet to suit digestion, makes scarecrows, and men and women who have no nerve although they may be all nerves. The latter, of toning up the digestion until it is equal to the necessities of the body, turns out men and women such as Shakespeare loved, and such as glorified "the spacious times of great Elizabeth." But, indeed, it needs little argumentation to show that monotony in eating and drinking must always fail to produce a high degree of health and cheerfulness, since monotony in anything, in work or play, or even in love and religion, tends to the production of a dulness which is often little less than deadly. As for spareness of diet, let those practise it who like it—the ascetic, the fanatic, and the man who has ruined his digestion by gluttony or drunkenness or both. But for the man who has responsible work to do, and to do every day, a generous and varied dietary, with, if need be, the judicious use of stimulants in some variety, is indispensable. If now and again opportunity offers for him to secure a change, even a feast, which leads to entire forgetfulness of self and the world and its worries for a period, it is all physiological gain. A generous life is mostly a healthy and successful life. Of course, it is open to a man who loves dissipation to use these doctrines foolishly, to abuse them. But The Hospital always takes for granted a certain amount of common sense and rightness of purpose in its readers.

### Old Age and Fatty Hearts.

A story told by Dr. G. W. Balfour, in his book on "The Senile Heart," well serves the double purpose of marking the practical progress which scientific medicine has achieved within the past fifty years, and of administering a much-needed word of comfort and encouragement to those numerous workers who, as age approaches, begin to feel uncomfortable and anxious sensations about the region of the heart. "Many years ago," says Dr. Balfour, "a gentleman of seventy-seven consulted me as to severe fainting fits to which he was liable. A distinguished consultant, since dead, had told him that these attacks were due to fatty degeneration of the heart, and that treatment would be of no avail. The heart's impulse was imperceptible, the sounds faint, but pure, the arteries firm, but neither hard nor tortuous; the urine was free from albumin, and of normal specific gravity. . . . I told the patient that experience had taught me that hearts supposed to be fatty were often only weak. . . . The result of treatment was a steady improvement in health and in force of heart-beat, and the patient lived to be ninety, and did not die of heart failure in the end, but from senile asthenia." To many people, "fatty heart" is a perfect bugbear. But this is what Dr. Balfour has to say about the diagnosis of the disease: "It is absolutely impossible to diagnosticate fatty degeneration of the heart. We may surmise its existence, but we can only be certain of its presence when we see it post-mortem." If many middle-aged and old men could but have this written deep upon the tablets of their consciousness, what loads would be lifted from their minds. Yet doctors of small experience roll out a diagnosis of fatty heart with sonorous satisfaction, unheeding that to many a trembling father of a family it is like the sound of the death-knell. On the question of treatment Dr. Balfour is equally decided. "We are often told," he says "that there is danger in treating a fatty heart. . . . Yet the result of treatment in the case recorded was a cure, proving that a heart supposed to be fatty was only weak, and that a life supposed to be over only wanted the fillip of a few minims of digitalis to carry it on to almost the extreme of human longevity." So true is it, even in scientific medicine, that a little experience and common sense outweigh many shiploads of mere abstract theorising.

### Influenza Ordinary and Extraordinary.

"Influenza" of the epidemic type has killed its thousands during the last few weeks. How many has ordinary influenza, the influenza which is more familiarly known as a "severe cold," at least disabled, if not killed? It is probable that common influenza—a severe cold—has in a large number of cases been the real delinquent, though *La Grippe* has had to bear the whole of the blame. The object of asking this question is to remind readers, by no means for the first time, what an important part the naturally moist climate of England bears in the production of diseases of the "chilly" variety; and how much more important still is the part played by the artificial climate which we have at length succeeded in manufacturing for the metropolis. We are all searching everywhere for the cause or causes of influenza. One cause, we are certain, is the chill damp of the English winter; another, and far more important cause, is the smoky and foggy envelope which surrounds the metropolis and prevents it getting more than about a third of its proper supply of sunshine. If influenza—ordinary influenza, at any rate—is to be effectually checked, we must do two things: improve our climate by diminishing smoke and fog, and train ourselves to the art of living more intelligently in what has been called the art of "dodging" the climate. Those who live in the sunnier parts of England, and who at the same time take care to keep themselves and their homes dry and moderately warm in the damp weather, suffer least from what we here speak of as ordinary influenza. A word to the wise ought to be enough.

